# The revised remote area nurse model of consultation

**DOI:** 10.1111/ajr.13195

**Published:** 2024-11-07

**Authors:** Sue Lenthall, Sabina Knight, Colin Watson, Lyn Byers, Fiona Cameron, John Wright, Sally West, Roianne West, Madeline Ford, Stuart Mobsby, Katie Pennington, Oluwatobi Ajayi

**Affiliations:** ^1^ James Cook University Emerald Queensland Australia; ^2^ Flinders University Alice Springs Northern Territory Australia; ^3^ Nganampa Health Council Gawler West South Australia Australia; ^4^ Northern Territory Health Tennant Creek Northern Territory Australia; ^5^ University of Sydney Sydney New South Wales Australia; ^6^ Put Tasmainia before Australia CoHealth Bicheno Tasmania Australia

**Keywords:** Aboriginal and Torres Strait Islander Health, client consultation, remote area nurse, remote health

## Abstract

**Aim:**

The aim of this revision was to update the Remote Area Nurse (RAN) Model of Consultation (MoC) and was prompted by publication of the National Rural and Remote Nursing Generalist Framework (2013–2018), shifts in RAN workforce patterns, community health patterns and technology use.

**Context:**

Rural and remote residents face higher rates of hospitalisations, deaths and poorer access to health care with a significant burden of avoidable fatal conditions among Aboriginal and Torres Strait Islander peoples. Health care is mostly provided by RANs and Aboriginal and Torres Strait Islander Health Practitioners (ATSIHPs), addressing diverse health needs, a mobile population and navigating cross‐cultural situations. Despite challenges such as clinician shortages, RANs manage a significant portion of non‐emergency consultations. The RAN MoC was developed to ensure comprehensive, systematic and person‐centred care and to mitigate risk to the client, the nurse and the health service.

**Approach:**

The 11 expert panel members, all authors, revised the RAN MoC through a series of Microsoft Teams meetings, one face‐to‐face meeting and an exchange of emails. The principles were reorganised under the four domains of the National Rural and Remote Nursing Generalist Framework and mapped against the National Safety and Quality Primary and Community Health Care Standards.

**Conclusion:**

The revised RAN MoC is designed to provide evidence based culturally informed care, standardise RAN consultation best practice and improve the health outcomes of their clients. With the increased turnover and number of nurses ‘new’ to remote, more innovative approaches to education and dissemination of the model is necessary.

## 1 CONTEXT

In many very remote areas of Australia, health care is primarily provided by registered nurses (RNs), often referred to as remote area nurses (RANs), and Aboriginal and Torres Strait Islander Health Practitioners (ATSIHPs).^1^, ^2^ These health care professionals play a crucial role in delivering continuous, coordinated and comprehensive care to community members, addressing the diverse health needs of the community across the lifespan.^3^, ^4^ They manage acute and chronic health conditions, engage in health promotion and public health activities, while navigating cross‐cultural contexts characterised by divergent language and knowledge systems.^1^, ^2^, ^3^, ^4^ RANs face myriad challenges unique to their practice settings in rural and remote areas of Australia. These include a lack of physical resources, such as essential medical equipment, general pharmacy services, and access to specialists and allied health professionals. In emergencies, the absence of timely retrieval services can result in delayed treatment and adverse health outcomes.^1^, ^2^, ^3^, ^4^ High turnover rates are also a significant issue, with Northern Territory Health clinics experiencing a turnover rate of 148% between 2013 and 2015.^5^, ^6^ This instability is exacerbated by the reliance on agency staff with short‐term contracts,^5^ leading to a loss of continuity in patient care and increased workload for permanent staff.^7^

The shortage of full‐time equivalent (FTE) clinicians in very remote areas, and the lack of, and maldistribution of medical practitioners in Australia,^8^ magnifies the extended practice of RANs,^4^ including everyday non‐emergency consultations,^9^ estimated to be around 80% of their role according to the expert panel.

The RAN role in non‐emergency client consultations is a key distinction between mainstream registered nurses (RNs) and RANs. Most models of consultation are derived from general practice medicine. In a literature search for nurse consultation models, only specialised areas such as palliative and geriatric care were found.^10^, ^11^, ^12^

The original RAN Model of Consultation (MoC),^9^ introduced in 2003, was developed to support safe nursing practice in remote areas of Australia, addressing the complex needs of clients with high morbidity and chronic conditions. It emphasises holistic, systematic and culturally safe care, aiming to guide RANs in providing evidence‐based care.^9^

RANs traditionally used frameworks such as ‘SODA F’ to guide their assessment of clients^9^ (Table 1).^9^

**TABLE 1:** SODA F.

SODA F
**S**ubjective/story
**O**bjective/observations
**D**iagnosis
**A**ction
**F**ollow‐up^9^

One professional organisation for remote practitioners outlines four steps in an assessment process: (1) triage considerations; (2) collect data; (3) form a clinical impression; and (4) document and communicate it.^13^ However, these models often overlook the specific challenges of the target population and fail to promote health literacy or self‐reliance. Despite electronic patient record systems encouraging recalls and follow‐ups, the absence of a comprehensive consultation model limits their effectiveness in improving health outcomes.

It was contended that a more holistic MoC is required to mitigate mortality and morbidity rates for everyone, particularly Aboriginal and Torres Strait Islander people in remote areas. For example, when a client presents with a simple acute injury, the assessment and treatment of the injury is crucial. However, it is essential to recognise other health challenges the client may be experiencing in addition to their life circumstances, which may impact their health. While addressing the acute injury is necessary, neglecting undiagnosed conditions such as rheumatic heart disease and the living circumstances that contribute to ill‐health is likely to reduce the client's long‐term health status.^14^, ^15^

Recognising the evolving landscape, the expert panel emphasised the necessity of clearly articulating the values that underpin the model. Concurrently, there has been a shift in the nursing and midwifery professions, placing a heightened focus on cultural safety and culturally informed care.^13^, ^14^ The acknowledgment of racism and discrimination leading to adverse health outcomes and fatalities among Australia's Aboriginal and Torres Strait Islander peoples has gained traction.^16^ This awareness has resulted in the incorporation of cultural safety principles into the National Nursing and Midwifery Board of Australia's Code of Conduct for Nurses^17^ and the National Rural and Remote Generalist Framework.^18^

## 2 APPROACH

### 2.1 Original RAN model of consultation

The original version of RAN MoC was informed by seven principles: (1) culturally safe approach, (2) holistic and comprehensive, (3) systematic, (4) share power with the client, (5) provide coordination and continuity of care, (6) encourage clinical reasoning and (7) promote clinical safety and quality. The RAN MoC featured six steps: (1) open consultation; (2) history; (3) clinical examination; (4) assessment and discussion; (5) negotiate a management plan; and (6) close consultation.^9^ Implicit in the model and echoed in the Council of Remote Area Nurses of Australia (CRANA, now CRANAplus), Philosophy of Remote Area Nursing^19^ was the emphasis on respecting people's involvement in decision‐making about their health care, fostering self‐reliance through consultations and being cognisant of relevant public health issues. The CRANA Philosophy^19^ also acknowledged the historical impact on people's well‐being, applied a social justice lens to remote area nursing work and underscored advocacy and empowerment as critical nursing strategies to promote health choices and self‐determination.^18^ Despite serving as the overarching document guiding RAN practice, these aspects were not explicitly reiterated in the model.^9^

### 2.2 Revision of the model

The revised RAN MoC aims to significantly enhance health outcomes by providing evidence‐based, culturally informed care tailored to the unique needs of remote communities. Key intended outcomes include improved health metrics through the implementation of holistic and systematic care practices, which address both acute and chronic conditions.

For this revision, experts in remote area nursing with experience using the RAN MoC in practice, RAN educators and experts in remote among Aboriginal and Torres Strait Islander health were invited to form the expert panel. Twelve professionals were invited, and all accepted, with one unable to participate, resulting in a panel of 11 experts, all of whom, are authors of this paper.

In the first round of consultation, participants were presented with the principles of the RAN MoC and a copy of the National Rural and Remote Nursing Generalist Framework 2023–2027.^18^ A Teams meeting was conducted to discuss how to update the principles and align them with the framework. Although not all panel members could attend, notes were taken and distributed to all members after the Teams meeting. Panel members provided further feedback to the chief researcher via email. In Round 2, a draft of the principles, organised under Framework domains, was sent to the panel, along with a document mapping the principles against the National Safety and Quality Primary and Community Health Care Standards, highlighting identified gaps.^20^ The principles were revised during the meeting, and panel members sent additional comments and changes to the chief investigator via email. Round 3 took place at the 2023 CRANAplus conference. Before this face‐to‐face meeting, another draft of the principles and an update of RAN MoC steps were emailed to panel members. Subsequent rounds via email were conducted to finalise the model.

Table 2 of the revised RAN MoC outlines specific principles and steps that directly address the challenges of RAN practice and ensure that RANs are equipped to provide culturally safe and evidence‐based care, which is crucial in cross‐cultural communication and the management of complex health issues prevalent in remote areas.^9^, ^18^

**TABLE 2:** Remote Area Nurse (RAN) model of consultation.

Principles
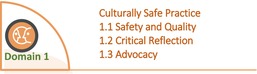
**Safety and quality**
Implements evidence‐based and strengths‐focused best practices in remote and Aboriginal and Torres Strait Islander health care Fosters a comfortable environment for the client through effective communication and cultural safety Addresses gender‐related considerations and assess the need for interpreter services Utilises appropriate language that respects cultural nuances Empowers the client by sharing decision‐making power and acknowledging their control over their own life
**Critical reflection**
Examine and reflect on how one's own culture and dominant cultural paradigms, influence beliefs about and interactions with Aboriginal and Torres Strait Islander peoples Recognise Aboriginal and Torres Strait Islander peoples' ways of knowing, being and doing in the context of history, culture and diversity, and affirm and protect these factors through ongoing learning in health care practice
**Advocacy**
Actively advocates for the client with fellow health professionals and other services
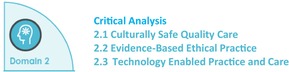
**Culturally safe quality care**
Explores client's experience of their illness, how it impacts them and their therapeutic goals Holistic, systematic and comprehensive Respects control over own life and encourages client to share in decision‐making Builds client's self‐reliance and health literacy Involves family as appropriate
**Evidence‐based ethical practice**
Guided by public health and primary health care principles Considers local disease patterns, and age/ place/risk Considers chronic diseases/ongoing health problems/age‐appropriate screening and preventative health care Recognises repeat presentations or an outbreak Use checklists/clinical references/clinical decision support tools
**Technology‐enabled practice and care**
Uses calibrated point of care testing, and tele‐imaging Uses telehealth to enhance diagnostics
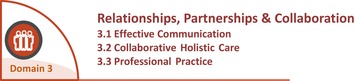
**Effective communication**
Negotiates and explains history taking, investigations and management plan options, including any factors impacting on care, such as transport, housing, and discusses findings Recognises the importance of comprehensive health record Clinical summaries to nominated services and to ‘my health record’
**Collaborative holistic care**
Recognises the importance of shared care Collaborates with colleagues, health and other services Builds partnerships with Aboriginal and Torres Strait Islander Health practitioners Coordinates complex care Responds to and activate recalls and follow‐up Documents comprehensively
**Professional practice**
Uses at least three patient identifiers Utilises clinical leadership
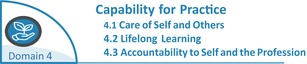
**Care of self and others**
Reflects on own and colleagues wellbeing Debriefs as appropriate
**Critical thinking**
Ability to critically think, analyse data and demonstrate clinical reasoning History (including past history) informs examination Considers what is most likely, clinically significant conditions and what you cannot afford to miss Rules in and out as required, including worst case scenario Identify red flags Recognises risk and the deteriorating patient
**Avoid diagnostic errors**
Premature closure—once you think you have the answer you stop looking Failure to consider all diagnostic possibilities Affective error—personal feelings (positive or negative) about a patient to affect clinical decisions Focusing on the presenting complaint and ignoring other health issues
**Accountability to self and the profession**
Takes accountability for personal and professional actions and decisions Seeks and responds to feedback to improve and develop professional capability for both self and colleagues Reflects on each consult and what has been learnt and what could be improved Actively works to enhance and promote multidisciplinary, rural generalist team Is aware of scope of practice and own ceiling of competence

Steps
**1. Open consultation**
*Before seeing client*
Review clinic records, note last visit and outstanding actions and review past investigations Get more information if needed Ensure room is comfortable, well lit, private Ensure appropriate equipment Consider own and client's safety
*Seeing client*
If not an emergency
Greet, establish rapport Note general impressions—appearance, speech, hearing, gait, posture, symmetry, tremors, odour, colour, skin, hands, mental state Observe interaction with others Check name, chart number, DOB, next‐of‐kin, need three identifiers Use appropriate language If appropriate, offer interpreter if necessary, involve family, offer gendered health professional if available
**2. History**
*Reason for presentation*
Acute/chronic disease review/screening
Story of why the client presented today Opening question ‘Why have you come in today?’ Use silence appropriately Establish concerns and expectations OLDCARTS (onset, location, duration, aggravating/relieving factors, treatments, associated signs & symptoms) Explain what you are doing/thinking, why you need to ask more questions Listen actively, encourage openness, do not interrupt Ask whether they been doing anything differently lately, travel work, activities If you cannot work it out—work backwards “what where they doing, what did they eat /drink that morning, last night, yesterday
*Current health status*
Appetite, nausea, change in weight Sleep, energy, physical activity Smoking/alcohol/other substance use—readiness to quit if indicated Urine, bowels, menstruation, sexual health Interest in life, emotional health/anxiety, self‐harm ‘Do you ever feel unsafe’
*Medicines*
Prescribed—what, when, how long, any problems Do they take medications as prescribed Over‐the‐counter, herbal, traditional, other people's medications Contraception (if appropriate)
*Allergies*—What happens when exposed, and how are allergies managed
*Immunisations*—Review status
**3. Clinical examination**
Rapid Physical Assessment including vital signs Screening tests as indicated including BGL, Hb, BMI, waist circumference, U/A, pregnancy test, ECG and other point‐of‐care tests Systematic assessment of relevant systems—LOOK, LISTEN, FEEL If client consents and timing appropriate, consider screening investigations as indicated by history, local epidemiology and age/place/risk
**4. Assessment and discussion**
Make an assessment of Reason for presentation Other health issues
Consider Age/Place/Risk—what is this client at risk of, considering their age and setting? What things are often missed/cannot be missed? What is most likely? Consult best practice treatment manuals Seek further advice remote medical practitioner or more experienced team member Summarise and reflect findings with client Explore client's knowledge and clarify misunderstanding—use ‘teach‐back’ method as appropriate Discuss long‐ and short‐term goals Assess and use opportunities for brief intervention and health promotion
**5. Negotiate management plan**
Review best practice treatment manuals for management guidelines Discuss goals/priorities including immunisation, negotiate management including referrals with client and significant others as appropriate Confirm management plan is acceptable to client and family, including what to expect, what do if gets better or gets worse Advocate for client as appropriate with health and other services Consider context and environment in negotiating management plan Plan follow‐up and long‐term management for identified risk factors, public health/preventive health issues and screening Ask if there are other questions, encourage and reassure Agree on follow‐up
**6. Close consultation**
Check client's understanding and agreement of management plan Provide appropriate illustrated or written material
**7. Documentation**
Be clear, legible, concise, contemporaneous, progressive and accurate Include information about assessments, action taken, outcomes, reassessment processes (if necessary), risks, complications and changes Meet all necessary medico‐legal requirements for documentation Update client record Enter information as appropriate into items in electronic system Follow the documentation style required by your organisation Refer as necessary for further investigations, support & management Update recall system Make use of Shared Electronic Health Record as appropriate and with consent
**8. Reflection**
How did the consultation go? What went well? What could have been done better? Identify own learning needs in relation to this condition/management, etc Consider your own self‐care and other colleagues
Permission has been granted from the Office of Rural Health to use the symbols for the four domains from the *National Rural and Remote Nursing Generalist Framework 2023–2027*

The RAN MoC principles were restructured and aligned with the four main domains of the National Rural and Remote Nursing Generalist Framework 2023–2027.^13^ Domain 1 Culturally Safe Practice^13^ includes the Capabilities Safety and Quality, Critical Reflection and Advocacy.^13^ Domain 1 was emphasised as a more explicit component of the RAN MoC. Strength‐based best practice^13^ approaches were incorporated into Safe Quality Care. The social determinants of health, as outlined by Wilkinson and Marmot,^21^ were more explicitly addressed with the addition of ‘Empowers the client by sharing decision‐making power and acknowledging their control over their own life’. Critical reflection includes examining and reflecting on one's own culture and recognising Aboriginal and Torres Strait Islander peoples' ways of being.^13^ The panel unanimously agreed that advocacy,^13^ though a significant part of any consultation, had been insufficiently articulated in the original MoC and was incorporated as a separate principle.

Domain 2, Critical Analysis, includes the Capacities of Culturally Safe‐Quality Care, Evidence‐Based Decision Making and Technology‐Enabled Practice and Care.^13^ In Culturally Safe‐Quality Care,^13^ a greater emphasis has been placed on building clients' self‐reliance and health literacy. Social determinants were also emphasised with ‘respects control over own life and encourages client to share in decision making’. Evidence‐Based Ethical Practice^13^ includes being guided by the principles of Primary Health Care, described as universal health coverage by building people‐centred, resilient and sustainable health systems that uphold the human right to health, promote social justice, empower individuals and communities, and address the determinants of health.^22^ It also includes public health principles, highlighting the need to consider local disease patterns and chronic diseases, age‐appropriate screening and preventative health care. Many of these principals were previously captured in the RAN Philosophy Statement^19^ and have been integrated into the revised model. Technology‐enabled practice and care was added to reflect the growing use of technology and telehealth in clinical practice.

Domain 3, Relationships, Partnerships and Collaboration includes the Capabilities of Effective Communication, Collaborative Holistic Care and Professional Practice.^13^ Effective Communication highlights the importance of the multidisciplinary team and shared care, including the use of clinical summaries and comprehensive health records. Collaborative Holistic Care recognises the importance of collaboration with colleagues, health services and other services. The importance of a partnership with Aboriginal and Torres Strait Islander Health Practitioners and professional practice were introduced.^13^ Professional Practice includes using at least three patient identifies as outlined in the National Safety and Quality Primary and Community Healthcare Standards,^20^ as well as clinical leadership.

Domain 4, Capacity for Practice includes the Capabilities of Care of Self and Others, Lifelong Learning and Accountability to Self and the Profession.^13^ Reflecting on one's own and colleagues' well‐being and debriefing was added to Care of Self and Others.^13^ The principles related to Critical Thinking,^13^ analysing data, and demonstrating clinical reasoning were reorganised with an additional section on avoiding diagnostic errors. Accountability to Self and the Profession^13^ emphasises the necessary to be accountable for personal and professional actions and decisions, seek feedback and being aware of the scope of practice and own ceiling of competence.

The model's steps, from opening the consultation to reflection, provide a clear, systematic approach to patient care, ensuring thorough assessments and continuous follow‐up. This structured approach not only improves the quality of care provided but also supports the professional development of RANs, ensuring they are well‐prepared to meet the diverse needs of their communities. By aligning with national health strategies and incorporating continuous feedback and updates, the revised RAN MoC represents a significant advancement in remote health care delivery, ultimately aiming to enhance health outcomes and community well‐being.

## 3 DISCUSSION

Anecdotally, it was previously customary for remote area nurses to commit to at least 2 years of service in a remote or very remote community. The RAN profession has long recognised that RANs are specialist generalists,^4^ and an undergraduate degree was considered insufficient for the unique RAN role.^9^ While various established courses, such as the Remote Health Practice program^23^ and a number of emergency courses,^13^ aim to prepare nurses to work as RANs, a significant portion of learning and teaching in this field occurred through interactions with more experienced colleagues, whose numbers are diminishing. The Australian Government's Northern Territory Emergency Response Commonwealth Intervention in the Northern Territory in 2007^24^ led to increased funding for short‐term visiting health workers, resulting in a rise in the use of agency staff.^12^ For instance, the Remote Area Health Corps (RAHC), established in 2008, received Commonwealth funding to recruit metropolitan‐based health professionals for periods of service ranging from three to 12 weeks.^5^ Consequently, many remote clinics now have few, if any, RANs employed directly by the health service.

The high turnover of RANs, workforce instability, and the prevalence of vacancies,^5^, ^6^ have contributed to an increase in nurses inexperienced in remote primary health care, a lowering of expectations regarding education and skill requirements, and additional stress and ‘orientation burnout’ for longer term RANs.^7^ A study exploring perceptions of RAN staffing in Northern Territory government health centres, concluded that RANs need advanced clinical and cultural skills, with Aboriginal staff and community members prioritising cultural skills and RANs and managers emphasising advanced clinical skills. Participants highlighted that having the right nurse was more important than simply having a nurse.^25^

Compelling evidence linking control over one's circumstances and health outcomes,^21^ strengthens the rationale for partnerships, shared decision‐making and active strategies that build self‐reliance. These evidence‐based contributions to improved health outcomes can and should be utilised by RANs in their consultation practice. Increasing the use of the RAN MoC^9^ aligned with the Rural and Remote Nursing Generalist Framework^18^ is considered a vital step to enhancing RAN practice both clinically and within a culturally safe framework. With the turnover and frequent use of short‐term staff, innovative models of education, wide dissemination and uptake by key organisations and health services are needed.

The broader implications of the revised RAN MoC for the community are substantial, with potential long‐term benefits that extend beyond immediate health care improvements. By emphasising culturally safe, holistic and systematic care, the model not only aims to address acute and chronic health conditions but also seeks to empower individuals and communities through enhanced health literacy and self‐reliance. Over time, this can lead to a significant reduction in health disparities, particularly among Aboriginal and Torres Strait Islander populations who have historically faced inequitable access to health care. The integration of culturally informed practices fosters trust and engagement between health care providers and community members, which is crucial for sustained health improvements.

## 4 CONCLUSION

The contemporary remote context, coupled with an increasingly under‐prepared nursing workforce, contributes to poorer health outcomes. The revised RAN MoC serves as a tool for RANs, enabling them to conduct holistic, systematic, evidence‐based, culturally informed primary care client consultations. This tool is designed for serving clients situated in remote regions of Australia, where there are challenges such as high morbidity rates, chronic diseases and multifaceted social, psychological, environmental and cultural requirements. The revised model stands as an instrument contributing to enhanced health outcomes in these challenging contexts.

Future directions for research and practice with the RAN MoC include a commitment to ongoing evaluation and continuous improvement. To ensure the model remains relevant and effective, regular feedback will be solicited from RANs and other health care practitioners using the model in various remote settings. This feedback will be systematically analysed to identify strengths and areas for improvement. As new evidence emerges and circumstances change, such as advancements in telehealth technology or shifts in community health patterns, the RAN MoC will be updated to reflect these developments. Collaboration with academic institutions, health care organisations and Indigenous communities will be crucial in this process, ensuring that the model evolves in a way that is both evidence‐based and culturally safe. By maintaining a dynamic and responsive approach, the RAN MoC aims to continually enhance the quality of care provided to remote and Indigenous populations, ultimately contributing to improved health equity and outcomes.

## AUTHOR CONTRIBUTIONS

**Sue Lenthall:** Conceptualization; methodology; writing – original draft; writing – review and editing; project administration. **Sabina Knight:** Conceptualization; writing – original draft; writing – review and editing. **Colin Watson:** Writing – original draft; writing – review and editing. **Lyn Byers:** Writing – original draft; writing – review and editing. **Fiona Cameron:** Writing – original draft; writing – review and editing. **John Wright:** Writing – original draft; writing – review and editing. **Sally West:** Writing – original draft; writing – review and editing. **Roianne West:** Writing – original draft; writing – review and editing. **Madeline Ford:** Writing – original draft; writing – review and editing. **Stuart Mobsby:** Writing – original draft; writing – review and editing. **Katie Pennington:** Writing – original draft; writing – review and editing. **Oluwatobi Ajayi:** Writing – original draft; writing – review and editing.

## CONFLICT OF INTEREST STATEMENT

There are no conflicts of interest.

## ETHICS STATEMENT

Not necessary.

## Data Availability

Data sharing is not applicable to this article as no new data were created or analyzed in this study.
